# Exploratory Algorithms to Aid in Risk of Malignancy Prediction for Indeterminate Pulmonary Nodules

**DOI:** 10.3390/cancers17071231

**Published:** 2025-04-05

**Authors:** Laurel Jackson, Claire Auger, Nicolette Jeanblanc, Christopher Jacobson, Kinnari Pandya, Susan Gawel, Hita Moudgalya, Akanksha Sharma, Christopher W. Seder, Michael J. Liptay, Ramya Gaddikeri, Nicole M. Geissen, Palmi Shah, Jeffrey A. Borgia, Gerard J. Davis

**Affiliations:** 1 Abbott Diagnostics Division, Abbott Laboratories, Chicago, IL 60064, USA; laurel.jackson@abbott.com (L.J.); nicolette.jeanblanc@abbott.com (N.J.); christopher.jacobson@abbott.com (C.J.); kinnari.pandya@abbott.com (K.P.); susan.gawel@abbott.com (S.G.); 2 Department of Anatomy and Cell Biology, Rush University Medical Center, Chicago, IL 60612, USA; claire_auger@rush.edu (C.A.); hita_moudgalya@rush.edu (H.M.); akanksha_sharma@rush.edu (A.S.); 3 Department of Cardiovascular and Thoracic Surgery, Rush University Medical Center, Chicago, IL 60612, USA; christopher_w_seder@rush.edu (C.W.S.); michael_liptay@rush.edu (M.J.L.); nicole_geissen@rush.edu (N.M.G.); 4 Department of Diagnostic Radiology, Rush University Medical Center, Chicago, IL 60612, USA; ramya_s_gaddikeri@rush.edu (R.G.); palmi_shah@rush.edu (P.S.); 5 Department of Pathology, Rush University Medical Center, Chicago, IL 60612, USA

**Keywords:** lung cancer screening, low-dose CT radiography, indeterminate pulmonary nodules, risk stratification, algorithm, circulating biomarkers

## Abstract

Background/Objectives: Lung cancer screening can reduce patient mortality. Multiple issues persist including timely management of patients with a radiologically defined indeterminate pulmonary nodule (IPN), which carries unknown pathological significance. This pilot study focused on combining demographic, clinical, radiographic, and common circulating biomarkers for their ability to aid in IPN risk of malignancy prediction. Methods: A case-control cohort consisting of 379 patients with IPNs (251 stage I lung tumors and 128 nonmalignant nodules) was used for this effort, divided into training (70%) and testing (30%) sets. Demographic variables (age, sex, race, ethnicity), radiographic information (nodule size and location), smoking pack-years, and plasma biomarker levels of CA-125, SCC, CEA, HE4, ProGRP, NSE, Cyfra 21-1, IL-6, PlGF, sFlt-1, hs-CRP, Ferritin, IgG, IgE, IgM, IgA, and Kappa and Lambda Free Light Chains were assessed for this purpose. Results: Multivariable analyses of biomarker, demographic, and radiographic variables yielded a model consisting of age, lesion size, pack-years, history of extrathoracic cancer, upper lobe location, spiculation, hs-CRP, NSE, Ferritin, and CA-125 (AUC = 0.872 in training, 0.842 in testing) with superior performance over the Mayo Score model, which consists of age, lesion size, history of smoking, history of extrathoracic cancer, upper lobe location, and spiculation (AUC = 0.816 in training, 0.787 in testing). Conclusions: In conclusion, a simple reduced algorithm consisting of biomarkers, clinical information, and demographic variables may have value for malignancy prediction of screen-detected IPNs. Upon further validation, this method stands to reduce the need for serial radiographic studies and the risks of diagnostic delay.

## Simple Summary

Annual low-dose CT-based lung cancer screening has been demonstrated to reduce patient mortality relative to chest X-rays, but has significant challenges in distinguishing malignant from non-malignant radiographic findings. To address this clinical need, we applied machine learning to readily accessible clinical information (i.e., patient demographics, clinical characteristics, and radiographic parameters) along with measurements from common circulating biomarkers to develop a method to aid providers with clinical decision making and reduce diagnostic delay inherent to current treatment standards.

## 1. Introduction

Lung cancer is the second most common cancer with >2.2 million cases annually diagnosed globally and is the leading cause of cancer mortality worldwide with >1.7 million deaths annually [1]. In the United States, non-small cell lung cancer (NSCLC) accounts for 80% to 85% of all lung cancers, with more than half of those patients diagnosed with advanced disease that is widely disseminated [2,3]. Patients who have been diagnosed with lung cancers that exhibit extensive metastatic progression have a 5-year survival of <10%, compared to approximately 60% for patients with localized disease [2,3]. These survival statistics highlight the need for an earlier-stage lung cancer diagnosis, where treatment initiation can yield superior patient outcomes.

In an effort to identify and treat lung cancer patients in the early stages of the disease, the National Cancer Institute’s National Lung Screening Trial (NLST) established that an annual low-dose helical computed tomography (LDCT) screening in specific high-risk groups reduces lung cancer fatalities [4]. Populations at a high risk of lung cancer that could benefit from this screening have been defined by the US Preventative Services Task Force (USPSTF) as apparently healthy patients between 55 and 75 years, who have at least a 20-pack-year smoking history and who currently smoke or have quit smoking within the past 15 years [5]. However, LDCT-based screening will not detect all lung cancers and often results in a high frequency of false-positive initial findings. LDCT false-positive findings can result in additional non-invasive and/or invasive procedures being performed to determine whether the observed nodule is malignant [4]. Typically, more than half of nodules found with increased LDCT screening of high-risk lung cancer populations are indeterminate pulmonary nodules (IPNs). IPNs are non-calcified lung nodules (typically 7–20 mm in size) that require further diagnostic workup for the risk of malignancy [6]. Pulmonary nodule size often correlates with greater risk of being cancerous: with nodules <5 mm: 0–1%; 5–10 mm: 6–28%; 11–20 mm: 37–64%; >20 mm: 64–82%; however, a variety of other factors including growth characteristics and spiculation can influence the ACR Lung CT Screening Reporting & Data System (Lung-RADS^®^) system for classification and nodule risk assessments [6,7]. Demographic data, such as age and smoking pack-years, have been combined with nodule size as aids in determining the risk of malignancy for IPNs. The model for determining the probability of malignancy in solitary pulmonary nodules, developed by the Mayo Clinic, is perhaps the most well validated [8,9,10] and is used in this report as a reference model. This six-parameter model (“Mayo score”) incorporates age, nodule size, current/former smoking history, extrathoracic cancer diagnosis ≥5 years prior, upper lobe location, and nodule spiculation using logistic regression to generate a probability of malignancy [8,10], but has not been shown to be sufficiently prognostic to resolve the clinical need [11,12]. As a result, additional diagnostic tools, such as circulating biomarkers, which may aid in further risk stratifying for malignant IPN, have become a topic of great interest in the scientific community [13,14].

To this end, this study explored the potential that the performance of risk-stratification models, such as the Mayo Score, could be enhanced upon the addition of circulating biomarkers. The following analytes were evaluated as part of this effort: CA-125, SCC, CEA, HE4, ProGRP, NSE, Cyfra 21-1, hs-CRP, Ferritin, total IgG, IgG1, IgG2, IgG3, IgG4, IgE, IgM, IgA, Kappa Free Light Chain, Lambda Free Light Chain, IL-6, PlGF, and sFlt-1, in addition to demographic variables such as age, sex, race, ethnicity, and smoking-pack-years. This study is an expansion of our initial pilot study with increased clinical cohort size, the inclusion of screening-detected nodule cases, and an expansion of patient variables to include in strengthening models toward more accurately assessing the risk of malignancy for patients with IPNs [8].

## 2. Materials and Methods

### 2.1. Sample Collection, Handling, Storage, and Immunoassay/Clinical Chemistry Testing

All subjects provided written informed consent prior to participation in this study, which was conducted under a protocol approved by the Rush University Medical Center (RUMC) Institutional Review Board (IRB #17071101/15021301). All subjects either met USPSTF guidelines for lung cancer screening and were recruited immediately prior to radiography or were treated by the Rush Department of Cardiovascular and Thoracic Surgery for a suspected lung malignancy and were recruited prior to surgical resection. The patient samples included in this study are reflective of the “real-world” population of patients who are screened or undergoing surgical resection for benign/malignant nodules at Rush University Medical Center in Chicago, IL. Peripheral bloods were collected by the Rush University Cancer Center Biorepository from each subject immediately prior to a clinical encounter (screening visit or anatomic resection) in lavender-top (K_2_-EDTA) vacutainer tubes using standard phlebotomy techniques. All peripheral blood specimens were handled and processed in an identical manner, with the time interval from venipuncture to processing being 90 min or less. Processing consisted of vacutainer centrifugation at 750 RCF for 20 min for initial plasma layer isolation, followed by a second centrifugation of the initial plasma isolates in a 15cc conical tube to remove residual particulates. All specimens were archived as 0.75 mL aliquots at 80 °C until use. No specimens were subjected to more than two thaw cycles for this study. All demographic and clinical parameters were linked to aliquots only in a coded (lacking patient identifying information) manner for evaluation. Tissue-based pathological assessments assigned by the Department of Pathology at RUMC relevant to the blood collection encounter were included in these reports for each case or, if unavailable, an account of the longitudinal radiographic behavior of the IPN, with stable or resolving nodules being classified as non-malignant [8].

After all samples were collected and shipped to Abbott, EDTA plasma levels of CA-125, SCC, CEA, HE4, ProGRP, NSE, Cyfra 21-1, IL-6, PlGF, sFlt-1, and Ferritin were determined by using the Abbott ARCHITECT^®^ i2000 automated immunoassay (IA) platform utilizing a two-step dual monoclonal chemiluminescent immunoassay [15]. The IL-6, PlGF, and sFlt-1 were Research Use Only (RUO) immunoassays. In addition, hs-CRP, total, IgE, IgM, IgA, Kappa Free Light Chain, and Lambda Free Light Chain were determined by using the Abbott ARCHITECT^®^ c8000 automated clinical chemistry (CC) platform utilizing a turbidimetric assay format (either utilizing antisera or latex-enhanced antibody-coated microparticles) [16]. The IA and CC platform testing determinations were performed at Abbott Laboratories (Chicago, IL, USA). Testing results were included in a database with the demographic and other clinical parameters.

### 2.2. Autoantibody Testing

Autoantibodies (AutoAbs) holding value for the early detection of lung malignancy, identified in previous work via microarrays or mass spectrometry were tested within the cohort [17,18]. Luminex assays were developed for the measurement of each of the AutoAb targets, upon covalent linkage of each of the recombinant protein targets to MagPlex beads, as previously described [17,18]. Assay ranges and standard curves were generated as previously described, using commercially available polyclonal antibody standards. A total of 14 novel single-plex/multi-plex assays for the measurement of 23 markers were produced and utilized to assess marker concentrations in the serum samples.

Serum samples were randomized and diluted 1:20 in assay buffer (1X PBS, 1% BSA, 0.01% Tween) prior to evaluations. All assays were performed in the 384-well format using a Bravo liquid handler (Agilent, Santa Clara, CA, USA), as previously described [18]. All plates were read on a FlexMAP 3D reader (Luminex Corp., Austin, TX, USA) with concentrations of each analyte calculated from the standard curves using the 4-PL logarithmic regression in Belysa v1.2 (MilliporeSigma, Burlington, MA). Only data with a minimum read of 25 beads/well and <50% coefficients of variation (%CV) were considered valid. Any marker measured with missing values >20% of the data was removed from the analyses. A full list of biomarkers tested is provided in Supplemental Table S1.

### 2.3. Statistical Methods

Cohort demographics were summarized by malignancy status for the present study. For continuous variables, median (IQR) and range were reported, and for categorical variables, number and percentage were reported. For markers with <20% of the data missing, values were imputed for all variables at once utilizing the missForest package v1.5, which makes use of a random forest algorithm to impute data via R version 4.0.2 [19].

Univariable analysis of the area under the curve (AUC) for malignancy was conducted for ARCHITECT^®^ platform immunoassay and clinical chemistry (IACC) biomarkers and the AutoAb biomarkers. To explore optimal combinations of biomarkers and clinical factors for malignancy discrimination, multivariable modeling was conducted. The cohort was randomly split into training (70%) and testing (30%) sets. Patients with a history of previous lung cancer (n = 29) were excluded from model development. As a comparative model to provide a baseline on this cohort, the solitary pulmonary nodule malignancy risk score (Mayo score) from the Mayo Clinic was evaluated on the training and testing sets [8,9,10]. This model incorporates age, nodule size, current/former smoking history, extrathoracic cancer diagnosis ≥5 years prior, upper lobe location, and nodule spiculation using logistic regression to generate a probability of malignancy [8]. In our analyses, the clinical variables evaluated corresponded to the predictors in the Mayo model, with pack-years used instead of current/former smoking history due to low rates of never-smokers in this dataset.

Using various combinations of clinical variables, IACC markers, and AutoAb markers, the logistic regression with the least absolute shrinkage and selection operator (LASSO) penalization method was employed to derive subsets of relevant variables in multivariable modeling. The tuning parameter lambda (λ) for the LASSO models was selected as one standard error from the minimum λ, using a 5-fold cross-validation approach [20]. A comparative model using a simple decision tree was also developed, with a candidate predictor set of clinical variables, IACC markers, and AutoAb markers using rpart (Recursive Partitioning and Regression Trees) in R, which implements the decision tree methodology outlined by Breiman and colleagues [21]. The complexity parameter for the decision tree was derived by evaluating a range from 0 to 0.10, choosing the value that maximized AUC across the 5-fold cross-validation. AUCs and corresponding 95% confidence intervals (CIs) were reported, along with sensitivity, specificity, positive predictive values (PPVs), and negative predictive values (NPVs) at a cutoff value of 50% predicted probability. Sensitivity at a fixed specificity of 90% and specificities at fixed sensitivities of 75% and 90% were reported as the median value across 2000 bootstrapped replicates. We then assessed the model limited to the screening of qualified patients, with performance evaluations conducted exclusively on the subset of patients that met USPSTF v2021 guidelines [5], as a supplemental analysis. All analyses were performed in R version 4.3.0 [22], and all biomarkers were transformed with logarithm base 2 for multivariable modeling.

## 3. Results

### 3.1. Patient Cohorts

The present cohort consisted of 351 patients, with 226 patients (64%) having a malignant nodule and 125 patients (36%) with IPNs classified as non-malignant. Patients with non-malignant IPNs were identified via two different pathways, those who were part of the Rush Lung Cancer Screening Program who had a radiographic LungRADSv2022 score of 3–4X at the time of sample collection and whose nodules were found to be stable (minimum 24 mo. radiographic follow up; median—88.4 months) or resolved upon consecutive scan (n = 45, 36% of non-malignant IPNs) or those who obtained a lung lesion biopsy at Rush and received a definitive histological diagnosis (n = 80, 64% of non-malignant IPNs). All patients with malignant IPNs were histologically diagnosed and staged by the Department of Pathology at RUMC (Table 1), with histological diagnoses provided in Supplemental Table S2.

### 3.2. Classification Algorithm Development

For model development purposes, the dataset was divided into training (n = 242, 68% malignant) and testing sets (n = 109, 57% malignant). Overall, patients in the training dataset had a median age (IQR) of 71 years (63, 75), with malignant patients being older on average compared to non-malignant patients (median 72 versus 66 years). Patient age in the testing set was comparable, with a median (IQR) of 68 (63, 75) overall, and 113 (47%) of patients in the train set and 54 (50%) of patients in the test set were male. In the training data, 207 (86%) of patients were white, 27 (11%) were black or African American, 3 (1.2%) were Asian, 1 (0.4%) was Native American or Pacific Islander, and 4 (1.7%) identified as other race, with comparable race characteristics in the test set. Smoking pack-years were lower on average for patients without malignancy, with a median of 40 pack-years for malignancy in both train and test sets, and 25 and 31 pack-years for patients without malignancy in the train and test sets, respectively. Median nodule size in training was 17 mm (IQR = 12, 26) and 16 (IQR = 11, 25) in testing, with malignant IPNs having higher nodule sizes on average compared to non-malignant. Compared to patients without malignancy, patients with malignancy also had higher proportions of nodules with upper lobe location (59% in malignant versus 42% in non-malignant in training) and spiculation (57% versus 36%), as shown in Table 1.

### 3.3. Classification Performance Characteristics of Algorithms

Univariable analysis of IACC and AutoAb markers found that IgM, hs-CRP, Cyfra 21-1, and TAF10 AutoAb had the highest observed single-marker AUCs in the training set (0.62, 0.62, 0.61, and 0.60, respectively) (Supplemental Figure S1). Multivariable analysis was performed to further improve the predictive power of biomarkers. Variable selection was performed on the training set using LASSO regression. For the four developed LASSO models (clinical variables only, AutoAb + clinical, IACC + clinical, and the full set of AutoAb + IACC + clinical), lesion size, upper lobe location, age, and pack-years appeared as predictors in each model. The full LASSO model (IACC + AutoAb + clinical) included age, lesion size, pack-years, history of extrathoracic cancer, upper lobe location, spiculation, IgE category ≥ 25 IU/mL, IgM, hs-CRP, CA-125, Ferritin, NSE, anti-TAF10 AutoAb, and anti-Ubiquilin-1 AutoAb (Table 2). According to LASSO variable importance coefficients, IgE category, upper lobe location, and IgM were the three most important variables in this model (Supplemental Figure S2). A comparative full model using decision tree methodology was also developed which included age, lesion size, pack-years, spiculation, hs-CRP, and Ubiquilin-1 AutoAb (Table 2).

The Mayo score model performed relatively well in the training set with an AUC of 0.816 (95% CI 0.758, 0.874) and similarly in the testing set (AUC 0.787; 95% CI 0.703, 0.872). This performance compares favorably to the report by Schultz et al. in 2008, where they observed a ROC of 0.80 (95% CI 0.72 to 0.88) in a cohort of 118 patients [8]. The LASSO clinical model resulted in a comparable included set of variables to the Mayo score except for a history of extrathoracic cancer (Table 2) and performed similarly to the Mayo score with an AUC of 0.824 (95% CI 0.769, 0.879) in training and 0.794 (95% CI 0.712, 0.876) in testing. The Mayo score was the most specific of all models (80.3% in training and 69.6% in testing) but with the tradeoff of a lower observed sensitivity (71.3% in training and 72.6% in testing) (Table 3). For reference, Supplemental Table S3 provides performance characteristics for the subgroup that meets USPSTF screening recommendations.

**Figure 1:**
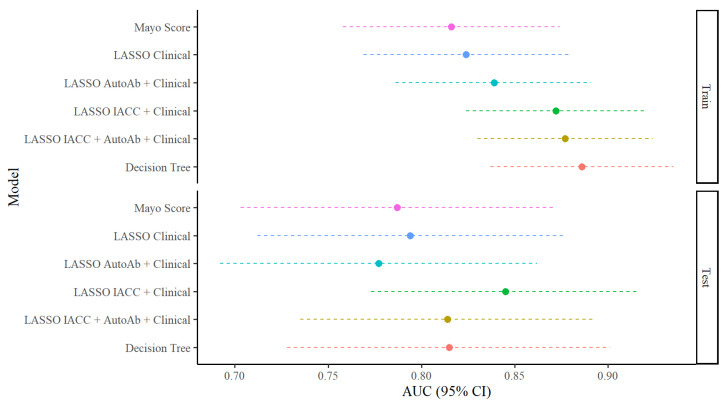
Area under the curve (AUC) of multivariable models for IPN category discrimination in train/test sets. AUCs and 95% confidence intervals (95% CIs) for 6 multivariable models (baseline Mayo score model, four LASSO regression models, and one decision tree model) for IPN category discrimination in the train and test sets.

In the training set, the addition of AutoAbs and IACC markers consistently increased AUC compared to the models with clinical variables alone, with higher increases occurring when IACC markers were included (Figure 1). In the testing set, this pattern held true apart from the LASSO AutoAb + clinical model, which had a lower overall AUC relative to the clinical models (see Figure 1; with ROC curves shown in Supplemental Figure S3).

**Figure 2:**
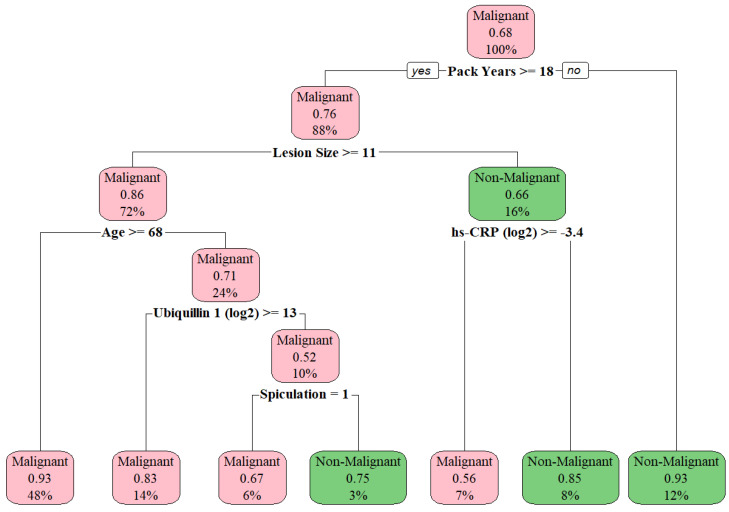
Decision tree model for IPN category discrimination illustrating the decision based on biomarker values or clinical factors at each split. Each box (node) indicates (1) the predicted class of the split (malignant/non-malignant), (2) the probability of the predicted class within that split, and (3) the overall percentage of patients in the node.

Overall, the decision tree model (which included IACC/AutoAb markers and clinical variables) had the highest AUC among evaluated models with an AUC of 0.886 (95% CI 0.837, 0.935) in training and 0.815 (95% CI 0.728, 0.901) in testing (Table 3). This model allows a simple schematic for interpretation, with the first split determined by pack-years ≥ 18 (model-derived cutoff), followed by further splits in the “yes” node for pack-years based on clinical characteristics and biomarker results (Figure 2).

The full model for LASSO including IACC/AutoAb markers and clinical variables demonstrated comparable performance to the decision tree, with an AUC of 0.877 (95% CI 0.830, 0.924) in training and 0.814 (95% CI 0.735, 0.893) in testing (Table 3).

## 4. Discussion

Current USPSTF lung cancer screening guidelines recommend annual LDCT scans for subjects with extensive smoking history and advanced age. LDCT scans are remarkably sensitive for pulmonary nodule detection [5,23]. Unfortunately, LDCTs lack sufficient specificity at baseline to permit IPN classification as malignant or non-malignant, creating a patient management issue. Approximately 10% of scans performed result in an IPN classification (LungRADs 3–4X). While rates can vary based on institution, approximately 14.6–18.6% of IPNs are eventually classified as malignant based on a diagnostic work-up [7,24]. For those with malignant nodules, valuable treatment time may be lost due to the need for follow-up, which can take up to 6 months for the LungRADs 3-score population. This is compounded by follow-up adherence being <50%; patients who initially presented at an early stage may end up with metastasis due to treatment delay [25]. In fact, one study reported that the median time between initial nodule identification to therapeutic resection was 98 days [26]. For patients with IPNs who are ultimately determined to have non-malignant lesions, costly follow-ups and procedures were required, which could lead to undue stress on the patient and potential mistrust of the screening process. These issues represent some of the major challenges for the current lung cancer screening paradigm.

Given these limitations of the current lung cancer screening standard of care, our study approach has been to risk-stratify radiological IPNs as malignant or non-malignant without the need for further follow-up LD-CT screenings to help guide optimal management of these patients. Demographic and clinical features have a well-recorded value within IPN evaluation, and there are current models, including the Mayo model, which is strictly based on such features [8,10]. In our study, this Mayo model served as a benchmark to highlight head-to-head the enhancements offered by the exploratory models developed in this study. This study explored the development of an IPN malignancy prediction model enhanced by the addition of common circulating biomarkers.

A key benefit of our approach was that we primarily included biomarkers that promise simplicity, accessibility, and low cost—given suitable clinical platforms and technology currently exist for their measurement. In general, platforms and regulatory paths are currently being developed to address tests that require digital health solution approaches for complex panels and algorithmic methodologies, such as the ones being piloted in this study. Clinical algorithms are not a new concept, with models such as those used for estimated glomerular filtration rate (eGFR) and calculations of low-density lipoprotein (LDL) levels being relatively commonplace within the clinical laboratory [27,28]. While specific, real-time reimbursement rates for tumor markers like CEA, CA 19-9, PSA, CA 125, CA 15-3, and Cyfra 21-1 vary based on insurance plans and locations, generally, reimbursement for these tests is common and often covered by insurance, with costs ranging from around USD 20 to USD 105 for individual tests, with clinical chemistry markers like Ig classes, CRP, and others noted as being even less expensive. With this, we would anticipate that the cost of a 5-test panel of existing clinical-use blood-based markers tested by immunoassay and/or clinical chemistry would be limited to a few hundred dollars.

In our study, all Abbott ARCHITECT^®^ IACC system clinical markers have been approved by the FDA for other clinical indications and are being explored for their value in nodule malignancy risk prediction. These markers are simple to measure and can, in some cases, be ordered as part of routine laboratory blood testing [29]. Individually, these biomarkers are inadequate for IPN risk of malignancy assessment, but, as part of an algorithm with clinical, radiological, and demographic information, may aid in enhancing IPN classifications to low- or high-risk malignancy categories. Immunoassay (IA) biomarkers evaluated included CA-125, SCC, CEA, HE4, ProGRP, NSE, Cyfra 21-1, hs-CRP, and Ferritin; they were chosen for their significant background in lung cancer screening algorithms research [7]. The Clinical Chemistry (CC) markers included were immune system profile markers IgG, IgE, IgM, IgA, Kappa/Lambda Free Light Chain, and IL-6. Changes in these immune system profile markers may reflect dysregulation of the immune system with early-stage lung cancer, which is a known hallmark of cancer [8]. Angiogenesis markers PlGF and sFlt-1 were selected for evaluation since angiogenesis is also considered a hallmark of cancer [8], and they have had promising performance characteristics defined by our group in a previous study [9]. The efficacy of the immune system biomarkers, angiogenesis markers, tumor markers, demographic variables, and clinical variables were explored as an aid in the classification of IPNs as likely non-malignant versus likely malignant.

In addition to the markers outlined above, we also included in this study an examination of AutoAbs, which can be produced when a B cell encounters a tumor-associated neoantigen, misfolded proteins, or aberrantly elevated antigens. Due to B-cell amplification, the production of AutoAbs compared to the initial antigen is often far greater. Additionally, AutoAbs exhibit greater stability within the circulation due to their size and endogenous production. Consequently, they have garnered significant interest as targets for early lung cancer detection, especially when serum antigen levels may not be markedly elevated due to a low tumor burden. The AutoAb biomarkers used in this study were repurposed from previous studies that demonstrated that the autoantibody targets may have utility in predicting “actionable” from “non-actionable” nodules at subsequent LDCT screening [18]. It was of interest to further assess these markers for the purposes of discerning non-malignant “actionable” nodules from malignant “actionable” nodules [17,18]. In this study, Ubiquilin-1 and the TAF10 AutoAbs were included in the final developed models. The Ubiquilin-1 AutoAb was determined to be a potential marker in our previous publication based on discovery via mass spectrometry [17]. Additionally, Ubiquilin-1 down-regulation has been implicated in the induction of phenotypic transdifferentiation (i.e., endothelial/epithelial to mesenchymal transition), cell viability, and cellular proliferation [17,30,31,32]. TAF10 AutoAbs were previously shown to be elevated in microarrays for patients with early-stage lung malignancy and high-risk lung malignancy patients. TAF10 AutoAb was also included as part of the final random forest model our laboratory developed for the identification of actionable pulmonary nodules [18]. The TAF10 protein has been linked to the transcriptional activation of MYC resulting in over-expression in cancer cells [33].

This study also found that circulating biomarkers enhance the test performance of patients with IPN beyond clinical features alone. Utilizing LASSO regression for feature selection, the top-performing combination of biomarkers was utilized to develop machine learning algorithms. We developed multiple models with improved AUCs compared to the Mayo model alone. In the training set, our top-performing model was the decision tree, which had AUCs of 0.886 and 0.815 in training and testing, respectively. In the testing set, the top performing model was the LASSO model comprising Abbott ARCHITECT^®^ IACC markers and clinical factors, with an AUC of 0.872 in training and 0.845 in testing. Given that the testing set is a separate evaluation of model performance, the LASSO IACC and clinical factors model may be preferable to consider as this study’s top-performing model overall, although these models warrant further exploration and/or validation.

In addition to model performance, the implementation and customizability of models are important to consider. Decision tree methodology allows for an easily implementable, intuitive model that can be directly applied by comparing the patient’s biomarker and clinical variable values against the decision tree thresholds to determine their risk category for malignancy (likely malignant or not malignant). Conversely, the LASSO model requires a software-based implementation platform to calculate the patient’s probability of malignancy. Nonetheless, this model is still interpretable in that the variable importance can be assessed and interpreted to determine the relative contributions of biomarkers and clinical factors. Further, this model inherently offers more customization from a risk-stratification perspective compared to the decision tree. Since the LASSO model provides a distribution of predicted probabilities that range on a continuous scale from 0 to 1, a cutoff could be optimized on the training set to achieve a desired target sensitivity or specificity, which is a distinct advantage over the simpler decision tree. This study did not seek to optimize cutoffs for clinical use, but these tradeoffs of model types as they pertain to implementation and customizability should be considered in future work. Additionally, the LASSO model is built strictly using clinical features and biomarkers measured using the ARCHITECT system. As previously mentioned, the ARCHITECT markers have already been approved for measurement in CLIA-certified laboratories due to their use for other clinical indications. This means that clinical implementation of this model may be expedited, once software implementation and further algorithm validation have been established.

## 5. Conclusions

In conclusion, we reviewed the potential of machine learning algorithms built from demographic variables, clinical variables, and commonly measured blood-based biomarkers, for the risk of pulmonary nodule malignancy prediction for IPN patients. We identified multiple candidate models, including a decision tree model, which holds the potential to be a simple classification model, and a LASSO model, which provides a predicted probability that could be optimized for sensitivity and specificity. We demonstrated that the classification performance holds up within both the typical screening population and for rare patients who undergo lung biopsies for non-malignant lesions. It will be important to assess how these models perform in future validation tests, which will include multi-institution larger sample size collections that better mimic a screening intended use population. It is anticipated that models from this study will likely be refined further with additional variables/biomarkers.

## Figures and Tables

**Table 1:** Cohort demographics for the train and test sets by IPN category.

	Train Set n = 242 (68.9%)			Test Set n = 109 (31.1%)		
Characteristic	Non- Malignant n = 78 (32%)	Malignant n = 164 (68%)	Overall n = 242	Non- Malignant n = 47 (43%)	Malignant n = 62 (57%)	Overall n = 109
**IPN Clinical Classification ^1^**						
Radiographically non-malignant based on follow-up radiography	27 (35%)	0 (0%)	27 (11%)	18 (38%)	0 (0%)	18 (17%)
Histologically diagnosed non-malignancies	51 (65%)	0 (0%)	51 (21%)	29 (62%)	0 (0%)	29 (27%)
Lung malignancies	0 (0%)	164 (100%)	164 (68%)	0 (0%)	62 (100%)	62 (57%)
**Age**						
Median (IQR)	66 (60, 71)	72 (66, 76)	71 (63, 75)	67 (60, 72)	71 (64, 77)	68 (63, 75)
Range	41, 82	44, 87	41, 87	43, 84	52, 87	43, 87
**Sex ^1^**						
Female	34 (44%)	95 (58%)	129 (53%)	22 (47%)	33 (53%)	55 (50%)
Male	44 (56%)	69 (42%)	113 (47%)	25 (53%)	29 (47%)	54 (50%)
**Race ^1^**						
White	63 (81%)	144 (88%)	207 (86%)	36 (77%)	54 (89%)	90 (83%)
Black or African American	11 (14%)	16 (9.8%)	27 (11%)	7 (15%)	5 (8.2%)	12 (11%)
Asian	2 (2.6%)	1 (0.6%)	3 (1.2%)	1 (2.1%)	2 (3.3%)	3 (2.8%)
Native American or Pac. Islander	1 (1.3%)	0 (0%)	1 (0.4%)	1 (2.1%)	0 (0%)	1 (0.9%)
Other	1 (1.3%)	3 (1.8%)	4 (1.7%)	2 (4.3%)	0 (0%)	2 (1.9%)
Unknown	0	0	0	0	1	1
**Ethnicity ^1^**						
Hispanic or Latino	1 (1.3%)	3 (1.8%)	4 (1.7%)	1 (2.4%)	0 (0%)	1 (1.0%)
Not Hispanic or Latino	75 (99%)	161 (98%)	236 (98%)	41 (98%)	61 (100%)	102 (99%)
Unknown	2	0	2	5	1	6
**Pack-years**						
Median (IQR)	25 (5, 43)	40 (30, 60)	40 (23, 50)	31 (3, 40)	40 (30, 50)	40 (30, 46)
Range	0, 120	15, 120	0, 120	0, 80	20, 150	0, 150
Unknown	1	2	3	0	1	1
**Nodule Size (mm)**						
Median (IQR)	12 (8, 18)	20 (14, 31)	17 (12, 26)	12 (8, 16)	20 (15, 31)	16 (11, 25)
Range	4, 53	7, 110	4, 110	6, 48	8, 95	6, 95
**Upper Lobe Location ^1^**	33 (42%)	97 (59%)	130 (54%)	23 (49%)	40 (65%)	63 (58%)
**Spiculation ^1^**	28 (36%)	94 (57%)	122 (50%)	18 (38%)	39 (63%)	57 (52%)
**History of Extrathoracic Cancer ≥ 5 Years Prior ^1^**	16 (21%)	44 (27%)	60 (25%)	12 (26%)	15 (24%)	27 (25%)

^1^ presented as n (%).

**Table 2:** Predictors from multivariable models for IPN category discrimination.

Model	Candidate Predictors	Final Model Predictors
**Mayo Score**	Age + Lesion Size + History of Smoking + History of Extrathoracic Cancer + Upper Lobe Location + Spiculation	Age + Lesion Size + History of Smoking + History of Extrathoracic Cancer + Upper Lobe Location + Spiculation
**LASSO Clinical**	Clinical variables: Age + Lesion Size + Pack-years + History of Extrathoracic Cancer + Upper Lobe Location + Spiculation	Age + Lesion Size + Pack-years + Upper Lobe Location + Spiculation
**LASSO AutoAb + Clinical**	AutoAb markers + clinical variables *	Age + Lesion Size + Pack-years + Upper Lobe Location + TAF10 AutoAb (log2)
**LASSO IACC + Clinical**	IACC markers + clinical variables *	Age + Lesion Size + Pack-years + History of Extrathoracic Cancer + Upper Lobe Location + Spiculation + IgE ≥25 + IgM (log2) + Spiculation + hs-CRP (log2) + NSE (log2) + Ferritin (log2) + CA-125 (log2)
**LASSO IACC + AutoAb + Clinical**	IACC markers + AutoAb markers + clinical variables *	Age + Lesion Size + Pack-years + History of Extrathoracic Cancer + Upper Lobe Location + Spiculation + IgE ≥25 + IgM (log2) + hs-CRP (log2) + CA-125 (log2) + Ferritin (log2) + NSE (log2) + TAF10 AutoAb (log2) + Ubiquilin-1 AutoAb (log2)
**Decision Tree**	IACC markers + AutoAb markers + clinical variables *	Age + Lesion Size + Pack-years + Spiculation + hs-CRP (log2) + Ubiquilin-1 AutoAb (log2)

* Same clinical variables as those in the “LASSO Clinical” model.

**Table 3:** Performance of multivariable models for IPN category discrimination in train/test sets.

Model	AUC (95% CI)	SE	SP	PPV *	NPV *	SE (SP = 90%)	SP (SE = 90%)	SP (SE = 75%)
**Train Set Performance (n = 242 with 164 events)**								
Mayo Score	0.816 (0.758, 0.874)	71.3	80.3	88.6	56.5	48.8	52.6	72.4
LASSO Clinical	0.824 (0.769, 0.879)	98.8	29.5	74.7	92.0	50.0	48.7	74.4
LASSO AutoAb + Clinical	0.839 (0.786, 0.891)	97.0	32.1	75.0	83.3	51.8	52.6	76.9
LASSO IACC + Clinical	0.872 (0.824, 0.921)	94.5	56.4	82.0	83.0	56.7	66.7	78.2
LASSO IACC + AutoAb + Clinical	0.877 (0.830, 0.924)	95.7	57.7	82.6	86.5	54.9	64.1	83.3
Decision Tree	0.886 (0.837, 0.935)	95.7	65.4	85.3	87.9	64.2	75.1	86.1
**Test Set Performance (n = 109 with 62 events)**								
Mayo Score	0.787 (0.703, 0.872)	72.6	69.6	76.3	65.3	45.2	41.3	69.6
LASSO Clinical	0.794 (0.712, 0.876)	96.8	27.7	63.8	86.7	53.2	46.8	59.6
LASSO AutoAb + Clinical	0.777 (0.692, 0.862)	91.9	25.5	62.0	70.6	53.2	36.2	59.6
LASSO IACC + Clinical	0.845 (0.773, 916)	87.1	61.7	75.0	78.4	59.7	53.2	74.5
LASSO IACC + AutoAb + Clinical	0.814 (0.735, 0.893)	90.3	53.2	71.8	80.6	56.5	53.2	68.1
Decision Tree	0.815 (0.728, 0.901)	91.9	66.0	78.1	86.1	28.8	68.1	70.7

* Positive and negative predictive values (PPVs and NPVs) are calculated at study prevalence (67.8% for the train set and 56.9% for the test set).

## Data Availability

Structured data is available upon email request from the corresponding authors.

## Supplementary Materials

The following supporting information can be downloaded at: https://www.mdpi.com/article/10.3390/cancers17071231/s1, with the following information: Supplemental Figure S1: Univariable analysis of markers for IPN category discrimination; Supplemental Figure S2: Variable importance from LASSO models; Supplemental Figure S3: ROC curves of multivariable models for IPN category discrimination in train/test sets; Supplemental Table S1: List of candidate AutoAb markers and IACC markers for multivariable modeling; Supplemental Table S2. Histological Breakdown of the Overall Cohort with Train and Test sets indicated. Supplemental Table S3: Subgroup performance of multivariable models among IPNs that meet USPSTF guidelines.

## Abbreviations

AUC	area under the curve
AutoAB	autoantibody
IACC	immunoassay and clinical chemistry, ARCHITECT^®^ platform
IPN	indeterminate pulmonary nodule
IQR	interquartile range
LASSO	least absolute shrinkage and selection operator
LDCT	low-dose helical computed tomography
Lung-RADS	Lung Imaging Reporting and Data System
Mayo score	solitary pulmonary nodule malignancy risk score from the Mayo Clinic
NLST	National Lung Screening Trial
NSCLC	non-small cell lung cancer
NPV	negative predictive value
ROC	receiver operating characteristic
RUO	research use only
RUMC	Rush University Medical Center
PPV	positive predictive value
USPSTF	US Preventative Services Task Force
