# Supplementary material for: Exploratory Algorithms to Aid in Risk of Malignancy Prediction for Indeterminate Pulmonary Nodules
Source: Cancers (Basel). 2025 Apr 5;17(7):1231. doi: 10.3390/cancers17071231 (PMC11988104; doi:10.3390/cancers17071231)
Supplement: Supplementary file 1 [file cancers-17-01231-s001.zip › cancers-3551548-supplementary.pdf]

Supplemental Figure S1: Univariable analysis of markers for IPN category discrimination. Receiver operating characteristic (ROC) curves and corresponding area under the curve (AUC) for IPN category discrimination for the top 10 biomarker predictors in the training set.

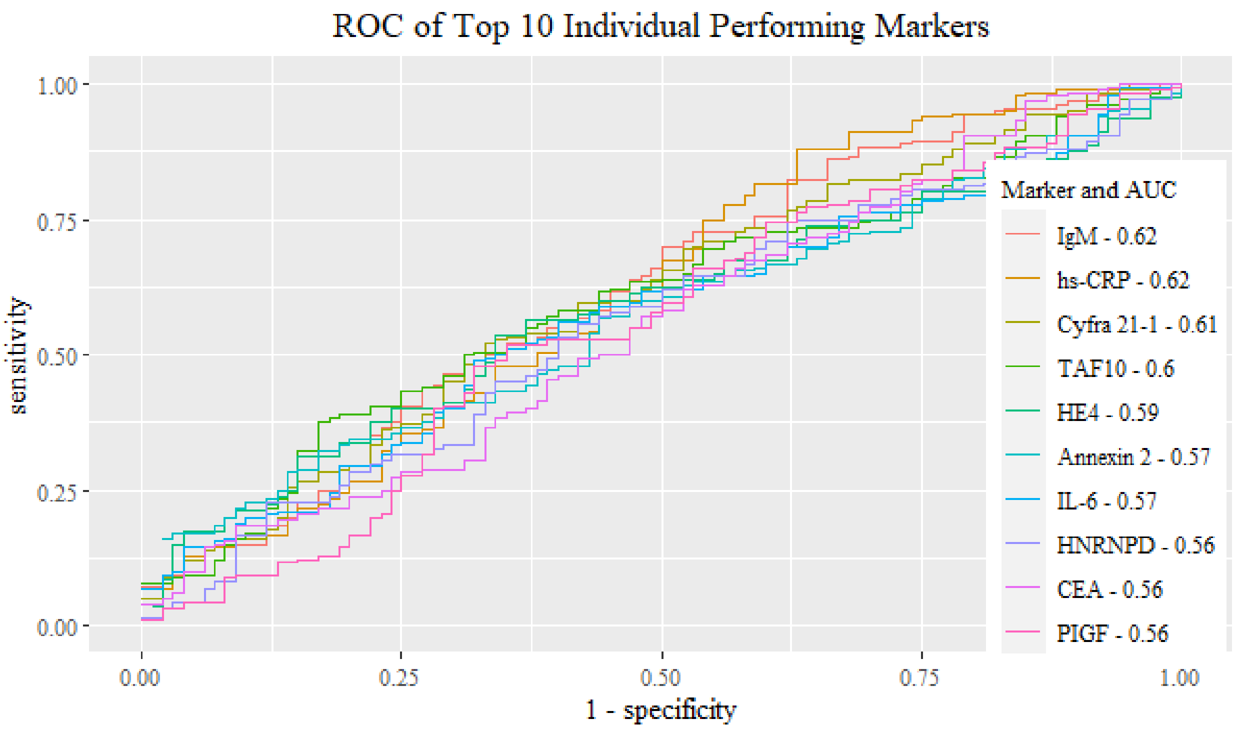

Supplemental Figure S2: Variable importance from LASSO models. : Variable importance reported as coefficients from logistic regressions with LASSO penalization method for IPN category discrimination using clinical factors only **(A)**, AutoAb and clinical factors **(B)**, IACC and clinical factors **(C)**, and IACC, AutoAb and clinical factors **(D)** as candidate predictor sets. Variables with a coefficient >0 are represented in light grey, and variables with coefficient <0 are represented in black.

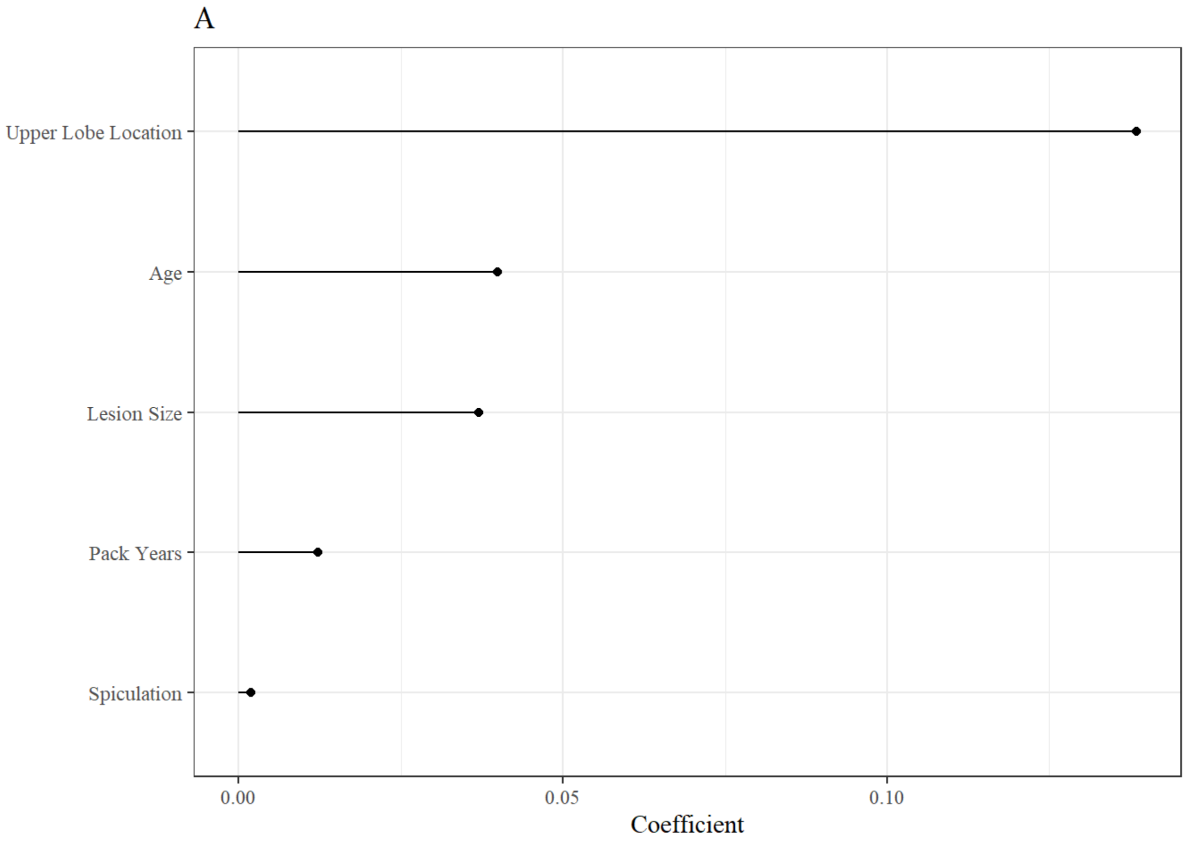

B

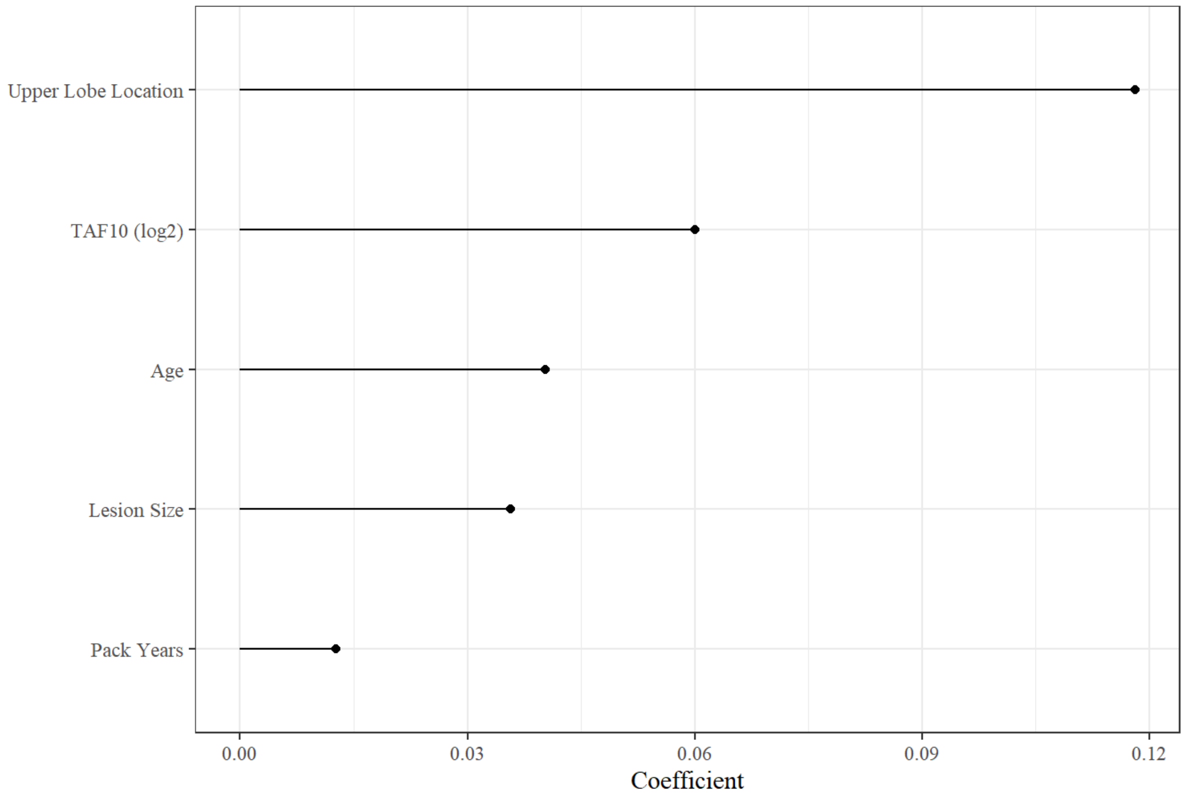

Upper Lobe Location
TAF10 (log2)
Age
Lesion Size
Pack Years
0.00
0.03
0.06
0.09
0.12
Coefficient

C

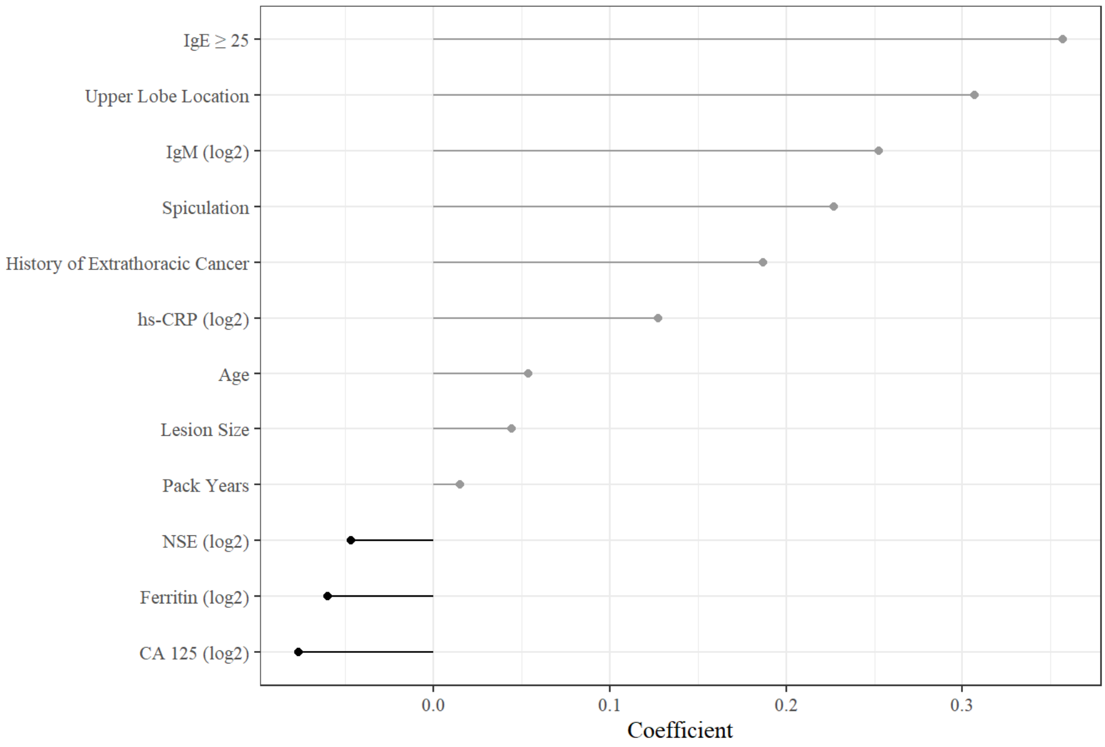

IgE ≥ 25
Upper Lobe Location
IgM (log2)
Spiculation
History of Extrathoracic Cancer
hs-CRP (log2)
Age
Lesion Size
Pack Years
NSE (log2)
Ferritin (log2)
CA 125 (log2)
0.0
0.1
0.2
0.3
Coefficient

D

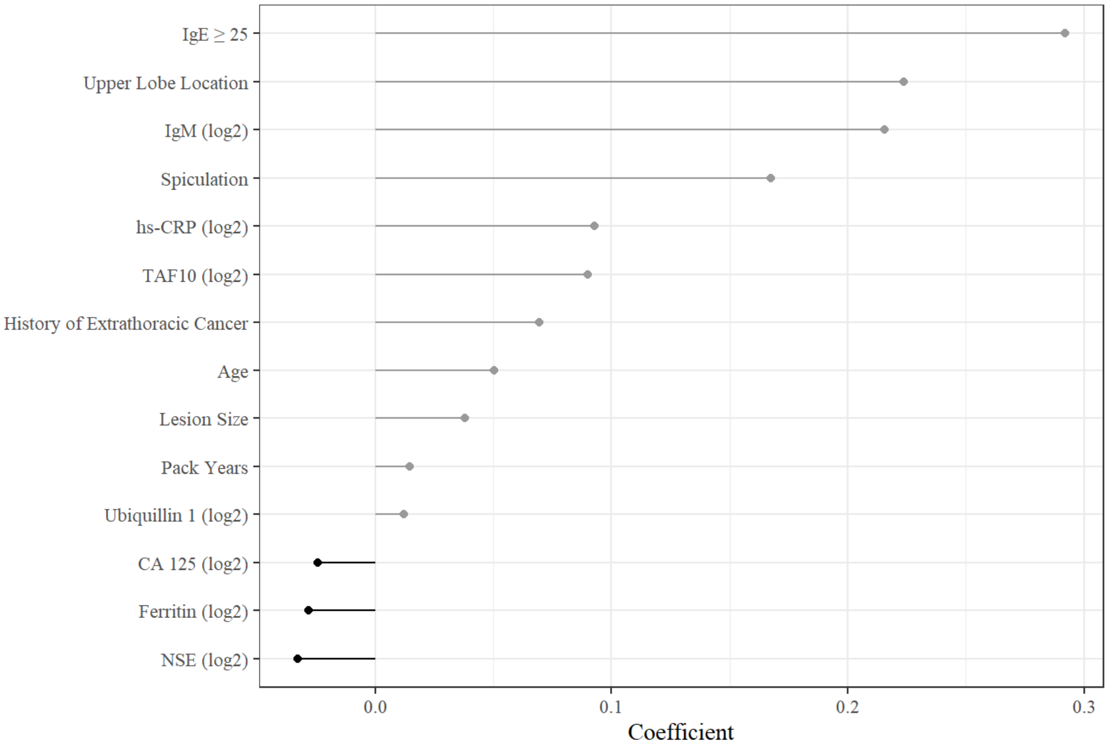
IgE ≥ 25
Upper Lobe Location
IgM (log2)
Spiculation
hs-CRP (log2)
TAF10 (log2)
History of Extrathoracic Cancer
Age
Lesion Size
Pack Years
Ubiquillin 1 (log2)
CA 125 (log2)
Ferritin (log2)
NSE (log2)
0.0
0.1
0.2
0.3
Coefficient

Supplemental Figure S3: ROC curves of multivariable models for IPN category discrimination in train/test sets. Receiver operating characteristic (ROC) curves for 6 multivariable models (baseline Mayo score model, four LASSO regression models, and one decision tree model) for IPN category discrimination in the train and test sets.

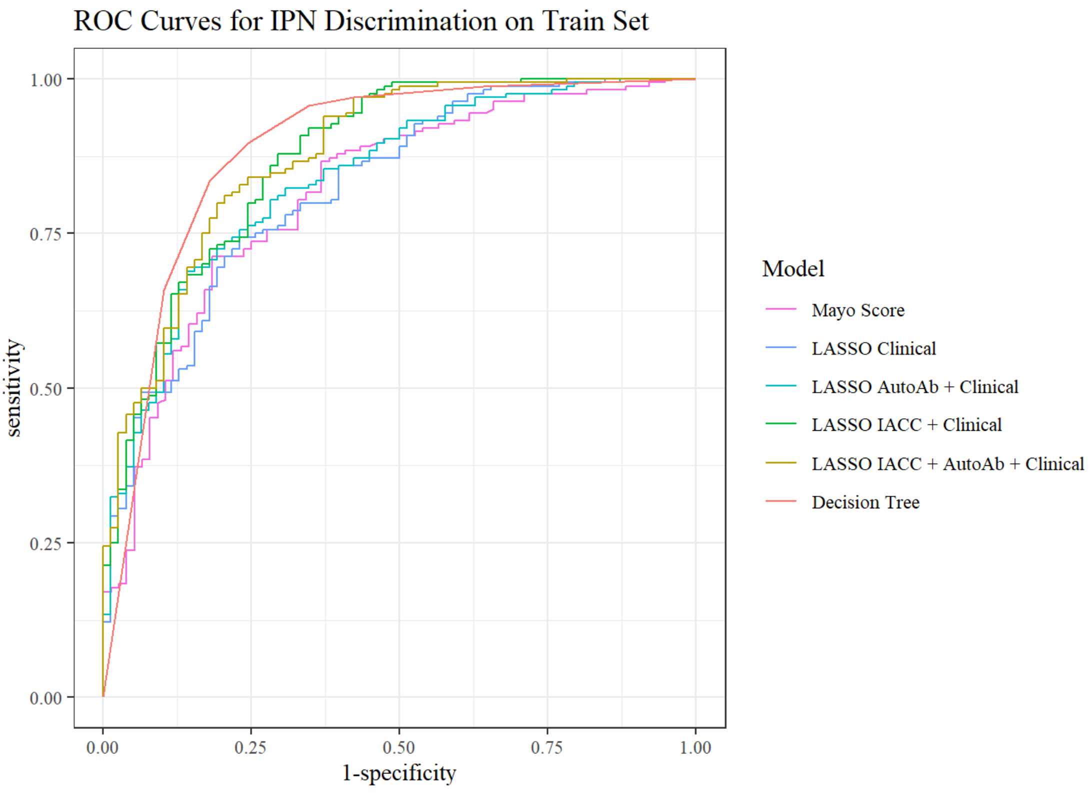

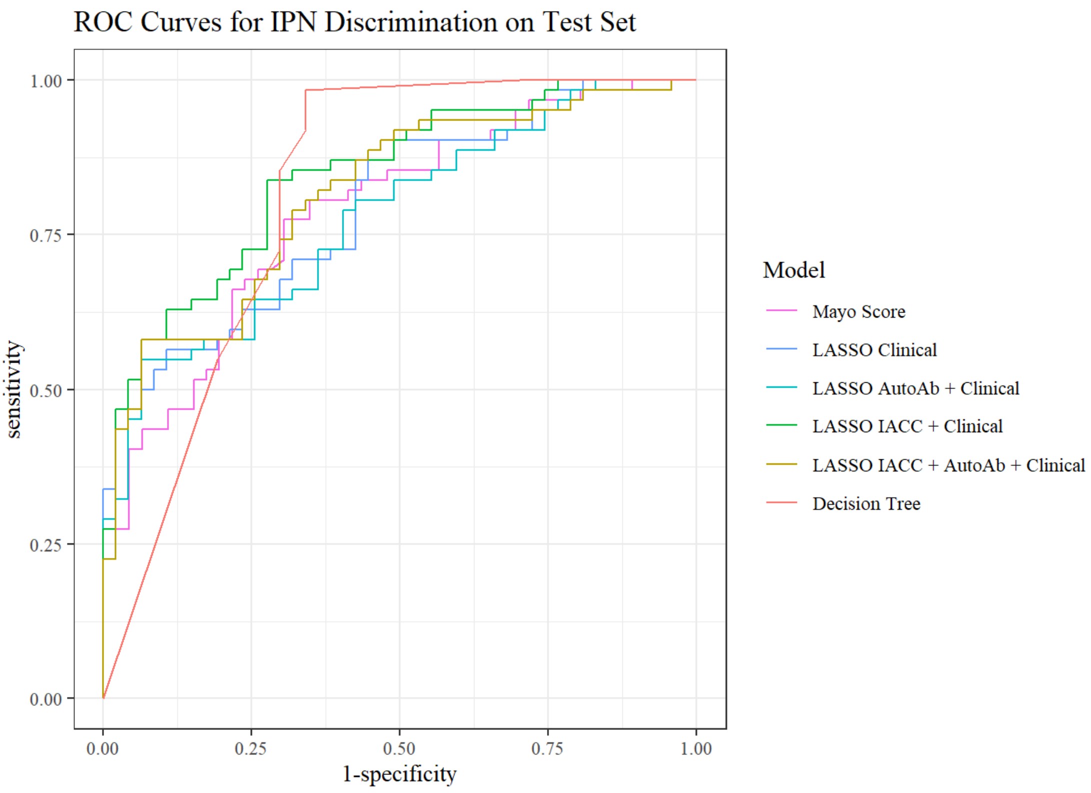

Supplemental Table S1: List of candidate AutoAb markers and IACC markers for multivariable modeling

Category	Included Candidate Markers
**IACC (Immunoassay/Clinical Chemistry)**	Cyfra 21-1, proGRP, SCC, CEA, NSE, CA-125, HE4, Ferritin, IL6, SFLT1, PIGF, IgG, IgM, IgE (categorized, ≥25), hs-CRP
**AutoAb (Auto-Antibody)**	Annexin 1, Annexin 2, Cilia and Flagella Associated Protein 36 (CFAP36), Dermcidin (DCD), Down-Regulator of transcription 1 (DR1), GC-rich Promoter Binding Protein 1 (GPBP1), Heterogeneous Nuclear Ribonucleoprotein D (HNRNPD), Heat Shock Protein 70 (HSP70), IKAROS Family Zinc Finger 5 (IKZF5), Inosine Monophosphate Dehydrogenase 2 (IMPDH2), Mediator Complex Subunit 21 (MED21), MID1 Interacting Protein 1 (MID1IP1), Myosin Binding Protein H (MYBPH), Nucleosome Assembly Protein 1 Like 5 (NAP1L5), Family with Sequence Similarity 192 Member A (FAM192A/NIP30), Phosphoglycerate Mutase 1 (PGAM1), Praja Ring Finger Ubiquitin Ligase 2 (PJA2), PNMA family member 1 (PNMA1), Sphingosine-1-phosphate lyase 1 (SGPL1), TATA-box binding protein associated factor 10 aa. 84-218 (TAF10), TATA-box binding protein associated factor 10, full-length (TAF10), Ubiquilin-1, Zinc Finger Protein 696 (ZNF696)

Supplemental Table S2. Histological Breakdown of the Overall Cohort with Train and Test sets indicated.

	Train Set n = 242 (68.9%)			Test Set n = 109 (31.1%)		
**Characteristic**	**Malignant** n =164 (68%)	**Non-Malignant** n = 78 (32%)	**Overall** n = 242	**Malignant** n = 62 (57%)	**Non-Malignant** n = 47 (43%)	**Overall** n = 109
Adenocarcinoma	89 (55%)	0 (0%)	89 (42%)	47 (76%)	0 (0%)	47 (52%)
Adenosquamous	2 (1.2%)	0 (0%)	2 (0.9%)	1 (1.6%)	0 (0%)	1 (1.1%)
Squamous cell carcinoma	56 (34%)	0 (0%)	56 (26%)	11 (18%)	0 (0%)	11 (12%)
Large cell/ Neuroendocrine CA	5 (3.1%)	0 (0%)	5 (2.3%)	1 (1.6%)	0 (0%)	1 (1.1%)
Carcinoid	4 (2.4%)	0 (0%)	4 (1.9%)	1 (1.6%)	0 (0%)	1 (1.1%)
NSCLC	3 (1.8%)	0 (0%)	3 (1.4%)	0 (0%)	0 (0%)	0 (0%)
Small cell carcinoma	4 (2.5%)	0 (0%)	4 (1.9%)	0 (0%)	0 (0%)	0 (0%)
Squamous Cell/Neuroendocrine	0 (0%)	0 (0%)	0 (0%)	0 (0%)	1 (1.1%)	1 (1.1%)
Granuloma	0 (0%)	20 (39%)	20 (9.3%)	0 (0%)	13 (44.4%)	13 (14.1%)
Hamartoma	0 (0%)	6 (12%)	6 (2.8%)	0 (0%)	5 (17%)	5 (5.5%)
Inflammation	0 (0%)	16 (31%)	16 (7.5%)	0 (0%)	9 (31%)	9 (9.9%)
Aspergilloma	0 (0%)	0 (0%)	0 (0%)	0 (0%)	1 (3.4%)	13 (14.1%)
Org. Pneumonia	0 (0%)	2 (3.9%)	2 (0.9%)	0 (0%)	0 (0%)	0 (0%)
Pneumonia with IgG4 related disease	0 (0%)	1 (2.0%)	1 (0.5%)	0 (0%)	0 (0%)	0 (0%)
Sarcoidosis	0 (0%)	1 (2.0%)	1 (0.5%)	0 (0%)	0 (0%)	0 (0%)
Scar	0 (0%)	5 (9.8%)	5 (2.3%)	0 (0%)	1 (3.4%)	1 (1.1%)
Unknown	1	27	28	0 (0%)	18	18

Supplemental Table S3. Subgroup performance of multivariable models among IPNs that meet USPSTF guidelines.

Model	N	Events	AUC (95% CI)	SE	SP	PPV	NPV	SE (SP = 90%)	SP (SE = 90%)	SP (SE = 75%)
**Train Set Performance: USPSTF Guidelines Qualified**										
Mayo Score	156	115	0.769 (0.680, 0.859)	67.0	77.5	89.5	44.9	37.4	47.5	65.0
LASSO Clinical	156	115	0.707 (0.618, 0.796)	98.3	14.6	76.4	75.0	40.9	29.3	46.3
LASSO AutoAb + Clinical	156	115	0.738 (0.654, 0.822)	95.7	17.1	76.4	58.3	43.5	31.7	53.7
LASSO IACC + Clinical	156	115	0.781 (0.696, 0.866)	92.2	41.5	81.5	65.4	40.0	48.8	61.0
LASSO IACC + AutoAb + Clinical	156	115	0.803 (0.725, 0.882)	94.8	43.9	82.6	75.0	44.3	51.2	70.7
Decision Tree	156	115	0.831 (0.753, 0.910)	96.5	48.8	84.1	83.3	41.3	62.5	78.2
**Test Set Performance: USPSTF Guidelines Qualified**										
Mayo Score	65	41	0.776 (0.658, 0.894)	63.4	75.0	81.2	54.5	34.1	45.8	66.7
LASSO Clinical	65	41	0.693 (0.560, 0.826)	95.1	16.7	66.1	66.7	34.1	25.0	54.2
LASSO AutoAb + Clinical	65	41	0.658 (0.523, 0.792)	87.8	16.7	64.3	44.4	39.0	20.8	45.8
LASSO IACC + Clinical	65	41	0.767 (0.648, 0.886)	82.9	58.3	77.3	66.7	46.3	41.7	66.7
LASSO IACC + AutoAb + Clinical	65	41	0.705 (0.575, 0.835)	85.4	41.7	71.4	62.5	36.6	33.3	54.2
Decision Tree	65	41	0.778 (0.640, 0.915)	87.8	66.7	81.8	76.2	18.7	66.7	70.8
